# Proactive Control of Emotional Information in Adult ADHD

**DOI:** 10.5334/joc.502

**Published:** 2026-05-11

**Authors:** Anna Längle, Ulrich Ettinger, Stefan Duschek

**Affiliations:** 1 Institute of Psychology, UMIT Tirol, Eduard-Wallnöfer-Zentrum 1, 6060 Hall in Tirol, Austria; 2 Department of Psychology, University of Bonn, Kaiser-Karl-Ring 9, 53111 Bonn, Germany

**Keywords:** adult ADHD, cognitive control, proactive control, inhibition, manual face-word Stroop task

## Abstract

Proactive control refers to the biasing of information processing toward a task goal during anticipation of a relevant event. This study investigated proactive control of emotional information in adult attention-deficit/hyperactivity disorder (ADHD) using a manual face-word Stroop task. Individuals with ADHD and healthy controls (*n* = 51 per group) were selected from a large participant pool (*n* = 1,020) using the Adult ADHD Self-Report-Scale. Images of happy or anxious faces were presented, with the word HAPPY or ANXIOUS written across the faces, congruent or incongruent with facial expressions. Participants had to identify facial expressions while ignoring word meaning. To investigate contextual cue utilization, the proportion of incongruent trials was manipulated (75% vs. 25%) to generate mostly congruent (MC) and mostly incongruent (MI) contexts. Individuals with ADHD showed larger error rate and longer reaction time (RT) than controls. Context effects were reflected by a greater difference in error rate and RT between congruent and incongruent trials within the MC than the MI context. These effects were independent of the presence of ADHD. The findings suggest that the ability to use contextual cues to facilitate proactive cognitive control is preserved in adult ADHD, despite marked impairments in attention and processing speed.

## Introduction

Cognitive control refers to regulation of thoughts, actions, and emotions to enable goal-directed behaviors (Miller & Cohen, 2001). The dual mechanisms of control (DMC) model is an influential framework, according to which cognitive control operates via two distinct modes, i.e., proactive and reactive control (Braver, 2012). Proactive control involves a prospective mode of control, biasing information processing toward a given goal during anticipation of a behaviourally relevant event. Reactive control is activated after such an event has occurred and allows detection of possible interference with goal attainment and its resolution by correction mechanisms.

Impairments in cognitive control are considered relevant to the pathology of attention-deficit/hyperactivity disorder (ADHD) (Onandia-Hinchado et al., 2021). ADHD is among the most frequently diagnosed mental disorders in children (Kooij et al., 2019); in many cases, symptoms persist into adulthood, which is reflected by a prevalence of ADHD of approximately 3% in the adult population (Kooij et al., 2019). Due to its main symptoms of inattention and/or hyperactivity-impulsivity, and high comorbidity with disorders including depression, anxiety disorders and substance abuse, adult ADHD is associated with severe impairments in wellbeing and quality of life (Safren et al., 2010).

Numerous studies have documented impaired reactive control in adult ADHD. A recent meta-analysis revealed substantial evidence of reduced performance on the stop signal task in this disorder (Senkowski et al., 2024). In the stop signal task, reactive control is quantified as the ability to withhold an automatic response following a stop signal that occurs in a small portion of trials (reactive inhibition). In contrast, research into proactive control in adult ADHD is still inconclusive. Proactive control can be quantified using tasks in which contextual cues provide information about (probable) control requirements in upcoming trials. An EEG study based on a cued task-switching paradigm showed smaller event-related potentials related to cue processing in adult ADHD; however, facilitation of behavioral performance due to the cue was unaffected (Sidlauskaite et al., 2020). Using task-switching and Stroop paradigms, King et al. (2007) showed impaired task-set coordination in ADHD, which partially depended on inefficient task preparation. White (2007) reported reduced resistance to verbal, but not spatial, proactive interference in adult ADHD. Moreover, studies applying the AX continuous performance test, a cued choice reaction task frequently employed within the DMC framework, did not provide evidence of impaired proactive control in adult ADHD (Levy et al., 2018; van Dijk et al., 2014).

The present study investigated proactive control of responses to emotional information in adults with pharmacologically untreated ADHD. This approach was chosen due to the well-known emotional peculiarities in the disorder. These comprise, for example, high proneness to negative emotions, increased emotional instability and irritability and low frustration tolerance (Skirrow & Asherson, 2013). Moreover, aversive emotional states and use of maladaptive emotional regulation strategies are prevalent in adult ADHD (Soler-Gutiérrez et al., 2023; Skirrow & Asherson, 2013). Therefore, impairments in cognitive control may be particularly pronounced when it comes to the control of emotional information.

Based on the inconclusive knowledge about proactive control and the observed emotional alterations in adult ADHD, this study explored possible impairments in the utilization of contextual cues using a manual face-word Stroop task (Jia et al., 2023). In the task, the image of a happy or an anxious face was displayed and the word “happy” or “anxious” was written across the face, either congruent or incongruent with the facial expression. Subjects had to identify facial expressions while ignoring word meaning which, in incongruent trials, requires inhibition of the automatic response of word reading. To manipulate levels of proactive control, the proportion of congruent and incongruent trials differed between contexts (Jia et al., 2023). A mostly incongruent (MI) context creates the expectation that automatic behavior must be overcome in upcoming trials (high proactive control); a mostly congruent (MC) context fosters automatic behavior (low proactive control) and a heavier reliance on reactive control processes. Impaired proactive control, i.e. reduced utilization of cues predicting the probability of response inhibition in ADHD would be reflected particularly by poorer performance within the MI context. However, the ambiguity of existing studies on proactive control in ADHD (Levy et al., 2018; Sidlauskaite et al., 2020; White, 2007) does not allow for the formulation of a definite hypothesis; thus, the study was exploratory.

## Method

### Participants

The study is part of a larger research project of cognitive and emotional processing in ADHD (c.f. Längle et al., 2025). Its design involved the comparison of individuals with ADHD and a healthy control group. Sample size estimation was based on the studies into cognitive deficits and emotional alterations in ADHD described in the Introduction, most of which revealed small-to-medium effect sizes. Assuming an effect size (Cohen’s *f*) of .13, an alpha error of 5% and a beta error of 20%, power analysis with G*Power (ver. 3.1.9.7) (Faul et al., 2007) revealed a required sample size of 49 participants per group.

During an initial screening, students from regional universities and higher educational centers completed the Adult ADHD Self-Report-Scale (ASRS; German version by Mörstedt et al., 2016). Individuals from the highest and lowest 5% of the ASRS sum score distribution (percentile ranks > 95% and < 5%, respectively; 51 participants per group) were selected for participation. Based on this procedure, ASRS data from 1,000 prospective participants would theoretically have been required to reach the intended sample size of 50 per group. However, only those who met the following criteria were included in the final sample: (1) aged between 18 and 35 years; (2) good physical health; (3) absence of relevant mental disorders except ADHD; (4) no use of psychoactive drugs (including any medications to treat ADHD); and (5) available to participate in a testing session at UMIT Tirol – University of Health Sciences and Technology. Due to these exclusion criteria, a total of 1,551 individuals had to complete the online survey to generate a preliminary sample of 1,020 individuals, among whom 102 were finally selected for participation (51 per study group).

Group characteristics were as follows; ADHD group: 36 women, 14 men, 1 without gender indication, age *M* = 23.98 ± 3.94 years, duration of education *M* = 16.24 ± 2.74 years, ASRS sum score *M* = 59.08 ± 3.79 (range 55–70); control group: 37 women, 14 men; age *M* = 23.14 ± 3.48 years, duration of education *M* = 16.12 ± 2.18 years, ASRS sum score *M* = 10.73 ± 10.73 (range 0–16). All participants from the ADHD group, and none of those from the control group, fulfilled the DSM-5 criteria of ADHD, diagnosed via the Structured Clinical Interview for DSM-5 (SCID; German version by Beesdo-Baum et al., 2019). Four of the participants from the ADHD group were diagnosed with the Predominantly Inattentive type of ADHD (DSM code 314.00); 47 were diagnosed with the Combined Type (inattentive/hyperactive-impulsive) of ADHD (DSM code 314.01). Relevant mental disorders (except ADHD) were detected in none of the participants, confirming the screening. Details of the recruitment procedure and the applied inclusion and exclusion criteria are described in the supplemental material.

### Measures

In the manual face-word Stroop task, images of happy and anxious facial expressions (370 × 472 pixel, 50% women, 50% men), taken from the Karolinska Directed Emotional Faces battery (Lundqvist et al., 1998), were presented against a black background using Presentation software (Neurobehavioral Systems, Inc., Berkeley, CA). The word HAPPY or ANXIOUS was written in red font across each face (font type: Arial, letter height: 1 cm), congruent or incongruent with the expression. Stimulus duration was 1500 ms; interstimulus intervals (white fixation cross) were 1900 ms, 2000 ms or 2100 ms (randomized, with equal frequency). The order of stimuli was identical for all subjects. Participants were instructed to indicate the facial expression as quickly and precisely as possible by pressing the left or right arrow key of a standard QWERTZ keyboard (assignment of keys to emotions counterbalanced across participants). In total, 148 trials were presented in two blocks, i.e. the MC and MI contexts. The MC context comprised 75% congruent trials and 25% incongruent trials; the MI context comprised 75% incongruent trials and 25% congruent trials (block order counterbalanced across participants). Happy and anxious expressions were equally frequent in both blocks and in congruent and incongruent trials. Task performance was indexed by error rate (in %) and reaction time (*RT* in ms) of correct responses.

### Procedure

Experimental assessment was conducted in a silent dimly lit room. Participants were requested not to drink alcohol on the day of the experiment or beverages containing caffeine for 3 hours prior to the session. The study was approved by the ethics board of the University of Innsbruck (Austria). All participants provided their written, informed consent.

### Statistical analysis

For statistical analysis, mixed ANOVAs were computed with the between-subjects factor Group (ADHD group vs. control group) and the within-subjects factors Congruency (congruent vs. incongruent trials) and Context (MC vs. MI context) using SPSS 27 software. Error rate and RT were dependent variables. Alpha was set at 5%; partial eta-squared ( $\eta_{\text{p}}^{2}$ ) is presented as effect size measure. ANOVAs were complemented by Bayesian analyses which allow for the probabilistic description of different hypotheses. JASP software was used for this purpose (Quintana & Williams, 2018).

## Results

**Figure 1:**
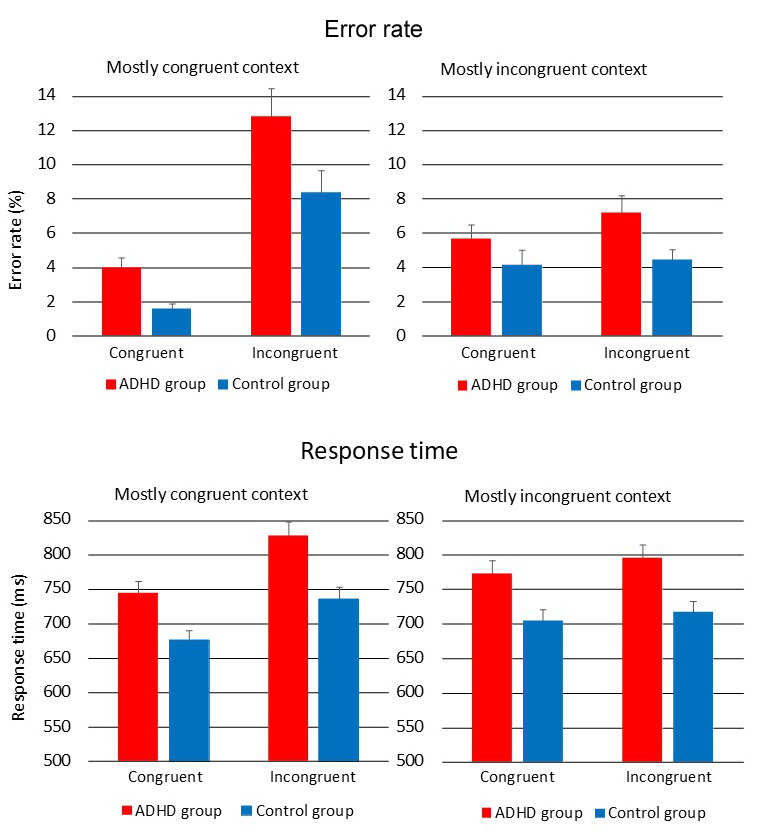
Error rate and reaction time in the ADHD and control groups for all experimental conditions.

Figure 1 displays error rate and RT for both groups and all experimental conditions. The ANOVA for error rate revealed a Group effect (*F*[1,100] = 8.80, *p* < .01, $\eta_{\text{p}}^{2}$ = .08), reflecting larger error rate in the ADHD group than in the control group. Congruency (*F*[1,100] = 64.48, *p* < .01, $\eta_{\text{p}}^{2}$ = .39) and Context (*F*[1,100] = 5.22, *p* = .02, $\eta_{\text{p}}^{2}$ = .05) effects indicated that error rate was higher for incongruent than congruent trials and for the MC than the MI context. Moreover, a Congruency × Context interaction arose (*F*[1,100] = 38.71, *p* < .01, $\eta_{\text{p}}^{2}$ = .28); the difference in error rate between congruent and incongruent trials was larger in the MC context than in the MI context. None of the further interaction effects were significant (Group × Congruency: *F*[1,100] = 2.18, *p* = .14, $\eta_{\text{p}}^{2}$ = .02; Group × Context: *F*[1,100] = 1.30, *p* = .26, $\eta_{\text{p}}^{2}$ = .01; Group × Congruency × Context: *F*[1,100] = 0.13, *p* = .72, $\eta_{\text{p}}^{2}$ < .01). Bayes analyses for error rate revealed a Bayes factor <. 001 for the Group × Congruence × Context model.

RT was longer in the ADHD group than in the control group (Group effect: *F*[1,100] = 11.36, *p* < .01, $\eta_{\text{p}}^{2}$ = .10). Moreover, RT was longer for incongruent than congruent trials (Congruence effect: *F*[1,100] = 102.27, *p* < .01, $\eta_{\text{p}}^{2}$ = .51); the RT difference between congruent and incongruent trials was larger for the MC context than the MI context (Congruency × Context interaction: *F*[1,100] = 41.43, *p* < .01, $\eta_{\text{p}}^{2}$ = .29). No further effects arose for RT (Group × Congruency: *F*[1,100] = 3.41, *p* = .07, $\eta_{\text{p}}^{2}$ = .03; Group × Context: *F*[1,100] = 0.17, *p* = .68, $\eta_{\text{p}}^{2}$ < .01; Group × Congruency × Context: *F*[1,100] = 0.73, *p* = .39, $\eta_{\text{p}}^{2}$ < .01). Bayes analyses for RT revealed a Bayes factor of 0.39 for the Group × Congruence × Context model.

Details of Bayesian analyses are presented in Table 1. For error rate and RT, the model with Group, Congruency and Context main effects, and a congruency × context interaction describes the data better than any alternative model. Considering the observed data, and based on the *a priori* likelihood of 5.3%, the probability of this model is 48.3% for error rate and 44.8% for RT. The factor value (BFm) exceeds 10 for error rate and RT, which supports the robustness of the model (Quintana & Williams, 2018). The result suggests that for error rate and RT the Congruency effect, the Context effect and the Congruency × Context interaction depend only to a very small extent on group membership.

**Table 1:** Results from Bayesian ANOVAs for correct responses and RT; P(M) = a priori probability; P(M|dat) = probability given the observed data; BF_M_ = Bayes factor; Only the best 10 models are displayed for both independent variables.


MODELS FOR ERROR RATE	P(M)	P(M|DAT)	BF_M_

Congruency + Group + Context + Congruency × Context	0.053	0.483	16.800

Congruency + Group + Context + Congruency × Group + Congruency × Context + Group × Context + Congruency × Group × Context	0.053	0.272	6.710

Congruency + Group + Context + Congruency × Group + Congruency × Context	0.053	0.115	2.329

Congruency + Group + Context + Congruency × Context + Group × Context	0.053	0.089	1.765

Congruency + Group + Context + Congruency × Group + Congruency × Context + Group × Context	0.053	0.025	0.456

Congruency + Context + Congruency × Context	0.053	0.017	0.314

Null model (including subject and random slopes)	0.053	<.001	<.001

Congruency	0.053	<.001	<.001

Group	0.053	<.001	<.001

Context	0.053	<.001	<.001

**MODELS FOR REACTION TIME**	**P(M)**	**P(M|DAT)**	**BF_M_**

Congruency + Context + Group + Congruency × Context	0.053	0.448	14.583

Congruency + Context + Group + Congruency × Context + Congruency × Group	0.053	0.311	8.120

Congruency + Context + Group + Congruency × Context + Context × Group	0.053	0.113	2.300

Congruency + Context + Group + Congruency × Context + Congruency × Group + Context × Group	0.053	0.083	1.639

Congruency + Context + Congruency × Context	0.053	0.024	0.436

Congruency + Context + Group + Congruency × Context + Congruency × Group + Context × Group + Congruency × Context × Group	0.053	0.021	0.389

Congruency + Group	0.053	<.001	<.001

Congruency + Group + Congruency × Group	0.053	<.001	<.001

Congruency + Context + Group	0.053	<.001	<.001

Congruency + Context + Group + Congruency × Group	0.053	<.001	<.001


*Note*. A priori probability denotes the prior probability assigned to the hypothesis before observing the data. In JASP, the average of all other models is used as a reference for BF_M_; the null model is used as a reference for BF_10_.

## Discussion

This study investigated cognitive control of emotional information in adult ADHD using a manual face-word Stroop task presented within MC and MI contexts. Individuals with ADHD showed markedly larger error rate and longer RT on the task than healthy controls. In the entire sample, incongruent trials were associated with larger error rate and longer RT than congruent trials. Context effects were reflected by a greater difference in error rate and RT between both trial types in the MC with respect to the MI context.

While emotional face expressions and emotional words are largely processed automatically, incongruence between both sources of information provokes a cognitive conflict (Beall & Herbert, 2008). This conflict must be resolved by applying cognitive control, which may involve inhibition of the response to the irrelevant source (Jia et al., 2023). As expected, increased load on cognitive control during conflict processing was associated with higher error-proneness and longer processing time in incongruent than congruent trials. The size of the effect of congruence on error rate and RT did not differ between individuals with ADHD and controls. In addition to ANOVA, this was confirmed by Bayesian analyses, suggesting that the congruence effect depended only to a very small extent on group membership. This observation is in line with studies applying the classical Stroop task (color-word interference test) in adult ADHD. According to a meta-analysis of 25 findings, color-word interference of children and adults with ADHD did not differ from that of controls without ADHD (Schwartz & Verhaeghen, 2008; however, see Lansbergen et al., 2007 for discussion of methodological challenges).

The MI context, within which incongruent trials are relatively frequent, allows anticipation and preparation of the cognitive conflict before the stimulus appears. Here, contextual information is thought to foster a sustained mode of proactive inhibition which increases response accuracy and reduces processing time in incongruent trials (Jia et al., 2023). In this study, this was reflected by large Congruence by Context interaction effects, indicating that the increase of error rate and RT in incongruent with respect to congruent trials was smaller during the MI context than the MC context. This is consistent with previously reported smaller color-word interference effect in the MI than MC context (Grandjean et al., 2012).

It is important to note that the Congruence by Context interaction for error rate or RT did not depend on the Group factor, suggesting that the interference effect varied according to context similarly in the ADHD group and the control group. In other words, individuals with ADHD did not differ from controls regarding their ability to use contextual cues to optimize task performance, thus successfully applying proactive control strategies. This was confirmed by Bayesian analyses, according to which the Congruency × Context interaction depended only to a very small extent on group status. This observation is striking insofar as the ADHD group displayed considerably reduced overall task performance both in terms of accuracy and speed. The increased error rate and RT that arose independent of trial type and task context likely reflects general impairments in attention and processing speed in adult ADHD. This accords with reports of deficits across a wide range of attentional functions in this disorder (see Onandia-Hinchado et al., 2021 for an overview). Within the MC context, incongruent trials appear infrequently and therefore somewhat unexpectedly, suggesting that reactive control may be activated to avoid mistakes. Adult ADHD was not associated with specific deficits in this ability, in contrast with previous reports (Senkowski et al., 2024). However, impaired reactive control in adult ADHD has mostly been observed in stop signal and go no-go tasks (Grane et al., 2016; Sebastian et al., 2012). These tasks mainly require interruption of automatic behaviors according to defined stimuli, such that their demands clearly differ from those of a paradigm based on context modulation (Jia et al., 2023).

In interpreting the findings, it should be noted that in tasks based on context manipulation, the impact of varying proportions of incongruent trials on congruency effects do not reflect cognitive control per se. Rather, individual differences in congruency effects relate to the degree of adaptation to these contexts. Therefore, the results suggest that in ADHD, the ability to appropriately adjust control to different contexts is preserved. While most previous studies on adult ADHD investigated cognitive control of neutral information, the present research was based on emotionally relevant stimuli. Facial expressions are essential to human interaction, as they allow rapid and automatic communication of emotional states and current needs (Duschek et al., 2025). Therefore, face stimuli are ecologically relevant, in addition to allowing investigation of the emotional alterations seen in adult ADHD (Soler-Gutiérrez et al., 2023).

A limitation of the study pertains to the task employed, in which levels of proactive control were manipulated via the proportions of congruent and incongruent trials. It has been demonstrated that the interaction pattern observed in such tasks may reflect contingency learning in addition to control demands (Schmidt, 2019). This confound can be mitigated by using tasks with four or more response options (Braem et al., 2019). Considering this, it must be acknowledged that the context effects observed in the two-choice task cannot be entirely ascribed to cognitive control (Spinelli & Lupker, 2023; 2024). Another restriction relates to the sample of young adults recruited from higher educational institutions, which may limit the generalizability of the findings to other populations. However, recruitment of an unmedicated sample allowed exclusion of effects of stimulants and other drugs used in ADHD treatment on cognitive processing. The exclusion of participants with psychiatric comorbidities limited distortion of the data due to cognitive and affective symptoms of disorders other than ADHD. A final limitation is that the study was not preregistered.

## Conclusion

This study extends our knowledge about cognitive control of emotional information in adult ADHD. Overall, performance in the manual face-word Stroop paradigm was strongly influenced by task context, as expected. However, whilst individuals with ADHD showed impaired response speed and accuracy, results suggest that the ability to use contextual cues to facilitate proactive cognitive control is preserved in the disorder.

## Additional File

The additional file for this article can be found as follows:

10.5334/joc.502.s1 Supplemental Material. Selection of participants.

## Data Availability

The research data of the study is available to the public via the repository Open Science Framework (OSF; https://osf.io/7mqan).
