# Supplementary material for: Proactive Control of Emotional Information in Adult ADHD
Source: J Cogn. 2026 May 11;9(1):29. doi: 10.5334/joc.502 (PMC13178611; doi:10.5334/joc.502)
Supplement: Supplemental Material. — Selection of participants. [file joc-9-1-502-s1.pdf]

Supplemental material: Selection of participants

Participants were selected according to the following steps. During an initial online screening, students from universities and higher educational centers in Tirol (Austria) completed the Adult ADHD Self-Report-Scale (ASRS; German version by Mörstedt et al., 2016). The online platform LimeSurvey (LimeSurvey, inc., Hamburg, Germany) was employed for this purpose. The ASRS consists of 18 items rated on a five-point Likert scale and provides scores for two subscales, termed Attention-Deficit (e.g., "How often do you make careless mistakes when you have to work on a boring or difficult project?") and Hyperactivity/Impulsivity (e.g., "How often do you fidget or squirm with your hands or feet when you have to sit down for a long time?"), in addition to a sum score. The Cronbach's α values obtained in the combined group (N=102) were .97 for the Attention-Deficit scale, .97 for the Hyperactivity/Impulsivity scale, and .98 for the sum score.

Individuals from the highest and lowest 5% of the ASRS sum score distribution (percentile ranks > 95% and < 5%, respectively) were selected for participation. Based on this procedure, ASRS data from 1,000 prospective participants would theoretically have been required to reach the intended sample size of 50 per group. However, only those who met the following criteria were included in the final sample: (1) aged between 18 and 35 years; (2) good physical health, determined using questions covering diseases of the cardiovascular, respiratory, gastro-intestinal and urogenital systems, the thyroid and liver, and metabolic diseases; (3) absence of relevant mental disorders except ADHD (e.g., affective and anxiety disorders, schizophrenia, obsessive-compulsive disorder or substance abuse), assessed via corresponding questions; (4) no use of psychoactive drugs (including any medications to treat ADHD); and (5) available to participate in a testing session at UMIT Tirol - University of Health Sciences and Technology. The criteria were assessed during online screening; moreover, to complete information, telephone interviews were conducted with all potential participants prior to their invitation to the testing session. Due to the described exclusion criteria, a total of 1,551 individuals had to complete the online survey to generate a preliminary sample of 1,020 individuals, among whom 102 were finally selected for participation (51 per study group).

References

Beesdo-Baum, K., Zaudig, M., & Wittchen, H. U. (2019). SCID-5-CV: strukturiertes klinisches Interview für DSM-5-Störungen-Klinische Version: deutsche Bearbeitung des Structured Clinical Interview for DSM-5 Disorders-Clinician Version von Michael B. First, Janet B. W. Williams, Rhonda S. Karg, Robert L. Spitzer. Hogrefe

Mörstedt, B., Corbisiero, S., & Stieglitz, R. D. (2016). Normierung der Adult ADHD Self-Report-Scale-V1.1 und der ADHS Selbstbeurteilungsskala an einer repräsentativen deutschsprachigen Stichprobe. Diagnostica, 62, 199-211. https://doi.org/10.1026/0012-1924/a000154
